# Combining BERT Model with Semi-Supervised Incremental Learning for Heterogeneous Knowledge Fusion of High-Speed Railway On-Board System

**DOI:** 10.1155/2022/9948218

**Published:** 2022-05-31

**Authors:** Lu-jie Zhou, Zhi-peng Zhao, Jian-wu Dang

**Affiliations:** ^1^School of Automation and Electrical Engineering, Lanzhou Jiaotong University, Lanzhou 730070, China; ^2^Key Laboratory of Railway Industry of BIM Engineering and Intelligent for Electric Power, Traction Power Supply, Communication and Signaling, Lanzhou Jiaotong University, Lanzhou 730070, China; ^3^Signal & Communication Research Institute, China Academy of Railway Sciences, Beijing 100081, China

## Abstract

On-board system fault knowledge base (KB) is a collection of fault causes, maintenance methods, and interrelationships among on-board modules and components of high-speed railways, which plays a crucial role in knowledge-driven dynamic operation and maintenance (O&M) decisions for on-board systems. To solve the problem of multi-source heterogeneity of on-board system O&M data, an entity matching (EM) approach using the BERT model and semi-supervised incremental learning is proposed. The heterogeneous knowledge fusion task is formulated as a pairwise binary classification task of entities in the knowledge units. Firstly, the deep semantic features of fault knowledge units are obtained by BERT. We also investigate the effectiveness of knowledge unit features extracted from different hidden layers of the model on heterogeneous knowledge fusion during model fine-tuning. To further improve the utilization of unlabeled test samples, a semi-supervised incremental learning strategy based on pseudo labels is devised. By selecting entity pairs with high confidence to generate pseudo labels, the label sample set is expanded to realize incremental learning and enhance the knowledge fusion ability of the model. Furthermore, the model's robustness is strengthened by embedding-based adversarial training in the fine-tuning stage. Based on the on-board system's O&M data, this paper constructs the fault KB and compares the model with other solutions developed for related matching tasks, which verifies the effectiveness of this model in the heterogeneous knowledge fusion task of the on-board system.

## 1. Introduction

The train control system incorporates various technologies, such as computers, control, and communication. It is the essential technical equipment for controlling train operations, ensuring operational safety, and improving operational efficiency. As an important component of the train control system, the on-board system plays a major role in operating and controlling the train. However, the on-board system works continuously for a long time, and the fault is inevitable. The fault has the characteristics of concealment and burst. At the same time, the monitoring data and maintenance data supporting the safe and reliable operation of the train control on-board system have the problems of multi-source heterogeneity and incomplete information. The main reason for these problems is that the sources of O&M data are complex, such as the manual records of drivers, railway experts, and maintenance personnel, as well as the equipment records of on-board vital computers, judicial recorder unit (JRU), and dynamic monitoring system (DMS), which constitute multi-source O&M record information [1]. At present, the maintenance of train control on-board system mainly depends on the technical staff to find out the causes of faults, formulate fault disposal measures, and complete on-board equipment fault information records or analysis reports. This method requires technical staff to repeatedly query and remember a large amount of fault information and maintenance knowledge. The fragmented and unorganized records make it difficult to share and inherit the fault analysis experience. Therefore, for complex and isolated data, it is necessary to use intelligent technology to extract knowledge from on-board fault maintenance records or reports to form a fault KB to provide support for the transformation of knowledge into a structured and visual knowledge graph. This method is of great significance for comprehensively mastering the key information of faults, realizing dynamic O&M auxiliary decision-making, and improving emergency response-ability. Due to the different data sources, forms, and publishers of on-board system fault KB, the expression forms of fault information are also different, resulting in semantic heterogeneity problems such as homonyms and homographs between knowledge units. The fault KB contains a lot of fuzzy and redundant information, which seriously affects the fusion of fault knowledge of the on-board system. Therefore, it is necessary to match the knowledge units in the KB, establish synonymous entity associations, and eliminate the inconsistency of knowledge expression to ensure the quality of the knowledge in the KB.a.

The fundamental strategy for dealing with the multi-source heterogeneity of knowledge units in on-board fault KB is to match the entities and realize knowledge fusion by judging whether different entities belong to identical objects in reality. The semantic ambiguity makes EM challenging due to the sparse knowledge unit representation and lack of context. Measuring the similarity of knowledge units is the key to the task of EM. The existing research on EM mainly adopts three methods: string similarity [2–4], structure similarity [5–8], and semantic similarity [9–13]. Traditional string-similarity-based methods focus on feature engineering and necessitate extensive theoretical knowledge from experts in order to accomplish EM by mining the similarity rules of knowledge units, which is hard to migrate to another domain. Structure and semantic similarity-based methods typically use knowledge embeddings and word embeddings to map entities in knowledge units into low-dimensional vectors by embedding representations, so that the semantic relevance of entities can be represented by the geometric structure of vector space. After that, various deep learning-based neural network models are used to complete the matching of heterogeneous knowledge units. Deep learning models can extract important features from embeddings automatically, thus avoiding complex feature construction. However, the network structure of neural network–based EM models should be carefully designed to capture the deep semantic or syntactic features of knowledge units to achieve optimal matching performance. In general, neural network performance is influenced by the training corpus, and the cost of corpus construction is very high in railway domain knowledge fusion tasks, where the scale of the domain corpus limits models' performance.

Although the early word embedding is trained on the corpus, it obeys the assumption of context independence, so each word has the same embedding after training. Pretrained models have recently become a research hotspot, such as BERT, OpenAI-GPT, ULM-FiT, etc. [14–17]. Such methods not only substantially improve the text's semantic representation ability but also facilitate model transfer applications, avoiding the burden of restarting the training after the model has been initialized. With relatively limited training data, BERT achieves competitive results in 11 natural language processing (NLP) tasks and significantly outperforms most embedding-based representations, such as word2vec and Glove. With intensive research on pretrained language models, these methods have achieved good performance in sentence matching, question answering, classification, etc. However, their potential in knowledge fusion tasks has not been fully explored. It is critical to make sufficient use of the limited supervised data in heterogeneous knowledge fusion tasks of the railway domain to fine-tune the BERT for task awareness and exact matching.

Aiming at the problem that the multi-source heterogeneity of on-board system O&M data affects the construction quality of fault KB, an EM model based on the combination of BERT and semi-supervised incremental learning is proposed, which formulates the fusion of on-board fault knowledge units as a task of pairwise binary classification of entities, to realize the fusion of multi-source heterogeneous knowledge. We investigate the advantages demonstrated by the proposed model for the task of heterogeneous knowledge fusion in high-speed railway on-board systems and design exhaustive experiments to assess our model's performance. This work consists of the following contributions:

1. For the multi-source heterogeneity of O&M data in high-speed railway on-board systems, a BERT-based EM model is proposed to extract deep semantic features from data-sparse and context-constrained knowledge units, and the impact of feature selection at different layers in the BERT model on the effect of heterogeneous knowledge fusion is explored.
2. To improve the utilization of unlabeled and limited-labeled samples, we propose a pseudo-label-based semi-supervised incremental training strategy that allows the model to collaboratively utilize pseudo-labeled samples to obtain higher knowledge fusion accuracy.
3. To strengthen the model's robustness to outliers and noisy data, we utilize an embedding-based adversarial training algorithm in the fine-tuning phase to update the model parameters by adversarial training on noisy data and clean data.

## 2. Related Work

### 2.1. Entity Matching

The existing research on EM mainly adopts three methods: string similarity, structure similarity, and semantic similarity.

The string-similarity-based method depends on complex feature engineering, such as entity character matching, attribute matching, or rule mining of knowledge units, and the effect of heterogeneous knowledge fusion is improved by carefully designing features. Such algorithms are effective at matching, but they are unable to handle textual heterogeneity, that is, knowledge units with different forms of expression [2]. In the task of constructing the KB of railway signal equipment, Li [3] proposed calculating the similarity between words in the railway domain through HowNet and then matching the knowledge units according to the set threshold and the similarity combination of words. In the multi-source heterogeneous knowledge fusion of steam turbines, Yan et al. [4] realized knowledge unit matching by combining character similarity and attribute similarity. This method has high requirements for knowledge unit standardization and requires the participation of a large number of domain experts, which is insufficient in universality.

The structure-similarity-based method relies on knowledge graph (KG) structural information to judge the equivalence of knowledge units. KG can be stored by < head entity, relation, tail entity > triples. These kinds of methods assume that entities in knowledge units representing the same real object in the knowledge graph have similar internal structural information. Through knowledge representation learning, entities or relationships in the KG are encoded to vector spaces, known as knowledge embeddings [5]. In this semantic space, those entities with identical or related meanings tend to be close to each other, and we can use knowledge embeddings to implement knowledge fusion. Existing KG embedding models include translational models [6] and deep models [7]. A recent work [8] points out that the artificially constructed KGs are denser than the real-world KGs. In the entity distribution of real knowledge graphs, most entities are only connected to one or two other entities, known as “long-tail entities.” In the structure-similarity-based knowledge fusion method, long-tailed entities have trouble drawing the attention of the model, so the method is not satisfactory in practice.

The semantic-similarity-based method is to transform the multi-source heterogeneity knowledge fusion task into a pairwise binary classification task of entities in the knowledge units and to realize the fusion of entity pairs with similar semantics through EM. Through the distributed representation technology, that is, word embedding, the entities of knowledge units are mapped from vocabulary to real-number vectors, which represent the semantic features of entities. Logical loss is then used to determine whether the word embeddings of the entity pairs match. For example, Kang et al. [9] first find the possible matching entity pairs by training knowledge embeddings using the KG structural information and use the Word2vec model to obtain word embeddings to select the final matching knowledge units based on the semantic-similarity model. In the literature [10], for the entity category matching task in geographic KB, the entities' semantic information is learned and represented as semantic vectors by word embedding methods, and the similarity in the entity categories is determined by calculating vectors so that geographic knowledge units can be fused. By training the fastText model, Zeng et al. [11] received independent word embeddings from the information about entity names, which were then combined with the information about entity structures to achieve knowledge fusion via iterative learning. Deep learning (DL) models are a focus of research in the current state of knowledge fusion based on EM. On-board system O&M data as text data can be converted into chain sequences, and the key to accurately extracting features from these chains is to capture the dependencies between adjacent elements, which often requires complex feature preprocessing or domain knowledge [12]. Mudgal et al. [13] reviewed and validated a variety of DL models for EM, including attention networks, recurrent neural networks, and smooth inverse frequency, as well as their variants, demonstrating the advantages of DL models in heterogeneous knowledge fusion tasks. Constructing entities of semantic matching models based on semantic similarity is an important method to realize multi-source heterogeneous knowledge fusion and has no special requirements for the scale of KB, which is more suitable for the fusion task of domain-specific KB. However, the knowledge units have a deficiency of lack of context, and it is difficult to represent the text features. In existing studies, word embeddings are usually obtained in an unsupervised way to represent the characteristics of knowledge units. This method does not consider the change in vocabulary context and maps entities into fixed vectors. The quality of vectors also directly affects the effect of downstream tasks.

### 2.2. Pretraining Models

Recent studies have proposed a method to pretrain the model through large unlabeled corpora and fine-tune it to implement the specific task to avoid complex task-specific model structure design and reduce the burden of learning parameters from scratch for the model in NLP tasks [14]. A three-stage model training approach is proposed in the transfer learning method ULM-FiT, consisting of a pretraining model, fine-tuning model, and fine-tuning classifier [15]. Some scholars have proposed the OpenAI-GPT model, which uses a large unlabeled corpus to train the model and learn general language representations, but the model is only based on unidirectional prediction [16]. Using past studies as a foundation, a BERT model based on deep self-attention was developed by Google scholars [17]. It is pretrained on the corpus by masked language model (MLM) and next sentence prediction (NSP), and then the model is fine-tuned using task-specific datasets so that no specific model structure is required, and the model performs well across in language processing tasks. BERT is built using deep bi-directional Transformer networks and pretrains the model with a large corpus, thus providing a deeper structural hierarchy and good parallelism. BERT can be used to better encode contextual representations and fine-tune specific downstream tasks.

Many innovative studies have been conducted using the BERT model. Tenney et al. [18] explored the syntactic and semantic structure within a sentence resolved by each layer of the BERT network. Based on their work, it has been demonstrated that basic syntactic features are typically extracted from the shallow structure of the model, and advanced semantic features are extracted from deep structures, and the use of advanced features helps disambiguate low-level decisions. Considering that each layer of BERT captures the different features of the input text, Sun et al. [19] investigated the effectiveness of features from different layers. By fine-tuning the different layers of BERT in the classification task, they observed that the feature from BERT's last layer gives the greatest results. At the same time, the last four-layer connection maximizes the collection of BERT information and achieves good performance. In GitHub's open-source bert-as-service [20] project, Dr. Xiao Han proposed that during BERT pretraining, the model's last layer will be closer to the predicted targets and the extracted features will be more skewed toward these targets. Therefore, this service works on the second-to-last layer of BERT. In this work, we combine high-speed railway knowledge and further apply BERT to the heterogeneous knowledge fusion task of on-board system.

## 3. Task Description of Heterogeneous Knowledge Fusion

**Figure 1:**
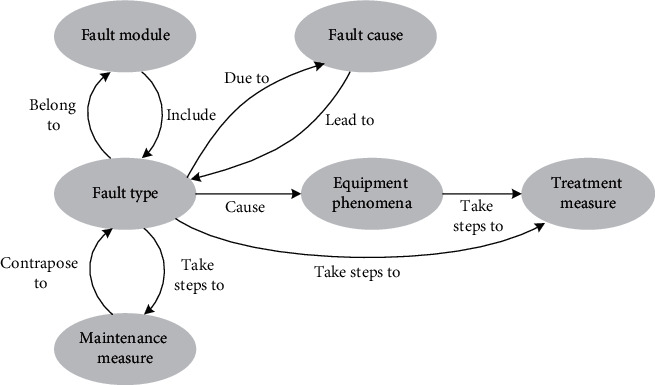
Fault knowledge data structure of on-board system.

To guarantee that the high-speed railway train control on-board system operates efficiently and reliably, the technicians of the railway electricity section and other relevant departments must monitor, analyze, and maintain the on-board equipment in time. Technicians locate the fault according to the operation data of the on-board system and formulate fault handling measures according to maintenance experience and form fault records. Therefore, during the O&M of the on-board system, a large number of unstructured records are accumulated, such as on-board system operation abnormality information analysis reports or tables, which record the operation status and maintenance of the on-board system in detail. Due to the lack of a uniform record format and data standard for on-board system O&M data, the data structure of fault records varies greatly. The problem of multi-source heterogeneity of data not only poses a challenge to the successful sharing of information but also results in an abundance of isolated data. Integrating multi-source heterogeneous on-board system Q&M data and establishing a uniform and standardized fault knowledge system for on-board equipment is the key to achieving intelligent Q&M decisions for the on-board systems. Therefore, we explore the construction of fault KB and multi-source heterogeneous knowledge fusion. Firstly, the key fault knowledge units are extracted from the multi-source heterogeneous data of the unstructured train control on-board system to construct the KB. Knowledge units primarily involve knowledge elements such as entities and relationships. Secondly, the heterogeneous knowledge in the KB is fused to ensure the quality of knowledge and provide support for the organization of knowledge units into a structured and visual KG. KG describes the relationship between faults and maintenance measures, which can help on-site technicians analyze the causes of faults and put forward maintenance suggestions. The construction of KB is a crucial step. The fault KB is mainly composed of entities such as fault modules, fault types, fault causes, fault analysis, equipment phenomena, treatment measures, and maintenance measures, as well as the relationship between entities. The data structure of the on-board system fault KB is shown in Figure 1.

The fault knowledge of the on-board system contains a large number of professional terms related to the railway field. Due to the different data sources, forms, and recorders of the fault KB, there are semantic heterogeneity problems of homonyms and homographs among entities, and knowledge units contain a large amount of fuzzy or redundant information. For further explanation, we present in Table 1 some examples of entities with the same meaning, i.e., entity 1 and entity 2 represent the identical real object. When combined with the characteristics of the entities in the O&M knowledge unit of the high-speed railway domain, the task of heterogeneous knowledge fusion will have the following three challenges:

Firstly, the challenge of a lack of context. It can be seen from the data types contained in the on-board system fault KB that the length of entities in the on-board knowledge units is short and the context is missing. It is difficult to obtain word embedding of entities in the case of sparse data and a lack of rich context. At the same time, there are too many similar characters between short entities in knowledge units, and the characters with differences are difficult to recognize. For example, when describing equipment maintenance measures, “Replace emergency brake relay” and “Check emergency brake relay” are two different maintenance measures, but they are only different in two Chinese characters. When describing equipment treatment measure, “Switching and restarting the system” can also be written as “Change system and reboot.” These two entities represent the same semantics but have more different Chinese characters. Therefore, it is difficult to judge the semantic difference between on-board fault knowledge only by the difference between characters.

Secondly, the challenge of data quality. The on-board system fault knowledge base contains a large number of professional terms. Due to the problems of format, unit, case, space, abbreviation nouns, typing errors, and so on, it will cause a lot of difficulties and interference in knowledge fusion. For example, “CTCS-3 exception downgraded to CTCS-2” can also be written as “C3 ⟶ C2” when describing equipment phenomena. In addition to the great differences in characters between the two entities, the problems of abbreviation and symbol substitution also affect knowledge fusion. Due to the different writing habits of technicians, some fault records are written in two versions, i.e., Chinese or English. When recording “wireless communication connection timeout” in the fault cause, “wireless” is mistakenly written as “infinite,” and this kind of homonym miswriting that leads to semantic ambiguity often occurs.

Thirdly, the challenge of obtaining a priori matching data. A priori matching data are also called training data. When the on-board fault knowledge fusion task is realized by the supervised method, a certain volume of labeled data is required. The number of on-board fault knowledge units is huge. If all entities in the fault KB are matched and labeled in pairs, the cost of construction is relatively high. Moreover, the limited-labeled data also limits the effect of fault knowledge fusion, and the unlabeled samples have not been fully utilized.

To eliminate the semantic conflict of knowledge units in the on-board fault KB and solve the problems of semantic heterogeneity of homonyms and homographs among entities, combined with the requirements of multi-source heterogeneous knowledge fusion in the railway field, the fault knowledge units are fused by constructing an EM model to ensure the quality of the on-board fault KB.

## 4. BERT-Based Model Knowledge Fusion

### 4.1. Data Preprocessing

In the fault KB of the train control on-board system, *K*=(*E*, *R*, *T*) is used to represent the knowledge, where *E* represents the entity, *R* denotes the relationship between entities, *T* represents triplet of the relationship facts of the entities in the fault KB. After defining the definition of the KB, we formally define the knowledge fusion task in the KB. Entities in the knowledge units can include any element in the fault KB. In particular, heterogeneous knowledge fusion in fault KB of train control on-board system can be defined as the matching task of entity pairs, and formally defined as:

(1) $$\begin{array}{c} M\text{atch}\left(K_{1},K_{2}\right)=\left\{\left(e_{1},e_{2}\right)|e_{1}\in K_{1},e_{2}\in K_{2}\right\}. \end{array}$$

The EM task is to find all similar entities and generate the matching result *S*={(*e*_1_, *e*_2_)*|e*_1_=*e*_2_, *e*_1_ ∈ *K*_1_, *e*_2_ ∈ *K*_2_}, with the equal sign indicating that the two entities represent the identical real object.

**Figure 2:**
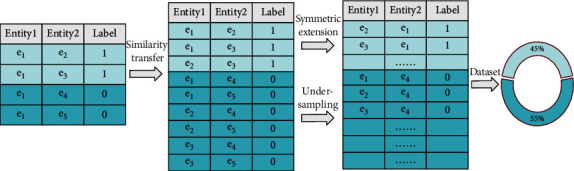
Construction method of the entity pairs data set of on-board fault knowledge.

The knowledge fusion task of train control on-board system fault KB is formulated into a binary classification problem of entity pairs in our work, and the heterogeneous knowledge fusion is completed by matching the semantic similarity between entities in the fault knowledge units. When constructing the entity pairs data set of on-board fault knowledge, according to the binary classification principle of fault knowledge matching, the matched entity pair is labeled as 1 and the unmatched entity pair is labeled as 0. We construct the data set according to the sample similarity transfer. To maintain the balance of the data set categories and reduce the influence of background samples, the symmetric expansion method is adopted in constructing the positive samples and the under-sampling method is adopted in constructing the negative samples. Samples that are positive will be labeled with 1, while samples that are negative will be labeled with 0. As shown in Figure 2, the construction method and scale of the on-board entity pair data set is illustrated. Entity1 and Entity2 represent the entities in two fault knowledge units, and Label is the tag of the entity pair.

### 4.2. Model Structure

**Figure 3:**
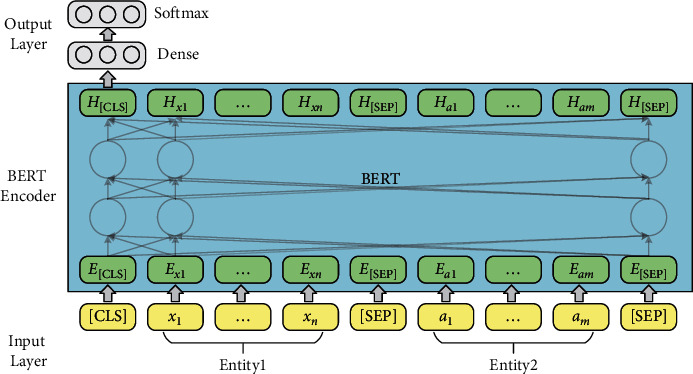
Structure of BERT-based model for EM for on-board systems fault knowledge.

We propose an EM model for train control on-board system fault knowledge based on BERT, which is used to realize the task of multi-source heterogeneous knowledge fusion.  Figure 3 describes an overall structure of BERT-based EM model for on-board system fault knowledge. The model components include the entity pair input layer for fault knowledge units, a BERT encoder, and the output layer for fault entity pair matching results. In the input layer, the entity pairs of fault knowledge units are used to construct the input sequence for the matching model. Then, the sequence of the input fault entity pair is encoded into a specific hidden state vector containing semantic information by the BERT encoder. Finally, the vector is transferred to the binary classifier to calculate the conditional probability distributions on the predefined fault entity pair categorical labels.

**Figure 4:**
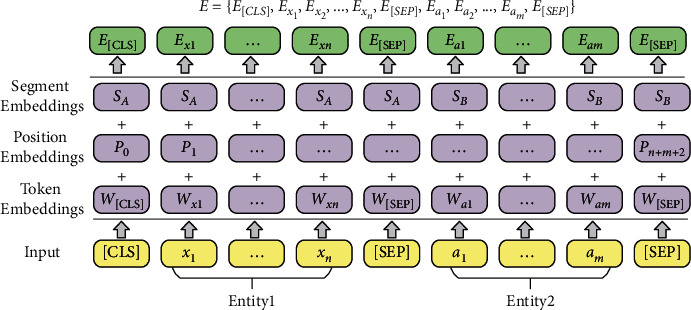
Construction of input sequence representations of the fault entity pair for BERT.

**Figure 5:**
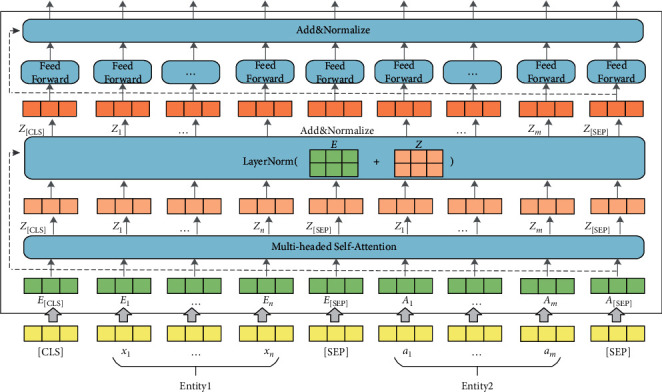
Basic structure of Transformer encoder.

**Figure 6:**
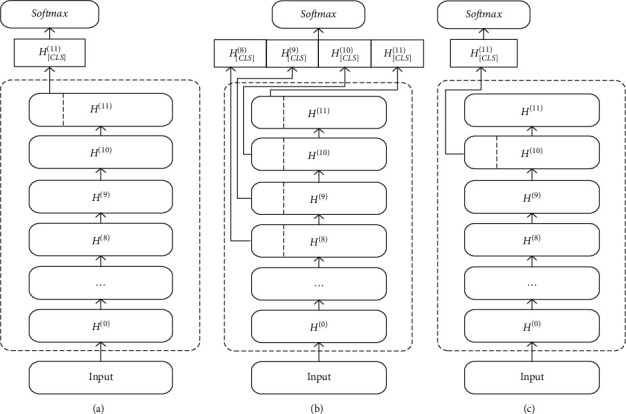
The output structure of the model (a) BERT_*LAST*_. (b) BERT_*CON*_. (c) BERT_*SEC*_.

(1) Input layer: The input sequence of tokens are entity pairs constructed using the entities in the on-board fault knowledge units, as shown in Figure 3. For an input entity pair *x*_1:*n*_={*x*_1_, *x*_2_,…, *x*_*n*_} and *a*_1:*m*_={*a*_1_, *a*_2_,…, *a*_*m*_}, we add a special token [CLS] to the beginning of the input sequences, and a special token [SEP] to the bottom of the input sequences and to the split between the two entities. The input sequence of on-board fault entity pair tokens is denoted as follows:
  (2) $$\begin{array}{c} I=\left\{\left[\text{CLS}\right],x_{1},x_{2},\ldots ,x_{n},\left[\text{SEP}\right],a_{1},a_{2},\ldots ,a_{m},\left[\text{SEP}\right]\right\}. \end{array}$$
For the input sequence *I*, the BERT model constructs token representations *E* as shown in Figure 4, primarily by the summation of the embeddings of the tokens *W*, positions *W*, and segments *S*. The token *a*_1:*m*_=(*a*_1_, *a*_2_,…, *a*_*m*_) representations of fault entity pair tokens are denoted as:
  (3) E=ECLS,Ex1,Ex2,…,Exn,ESEP,Ea1,Ea2,…,Eam,ESEP.
(2) BERT Encoder: BERT (Chinese version) has been pretrained on the Chinese Wikipedia corpora and is capable of extracting deep semantic features of common Chinese words. However, the on-board fault knowledge text for train control systems is very diverse compared to the common Chinese vocabulary, which contains special professional terms, language conversions, and abbreviations in the railway field. Therefore, we need to continue fine-tuning the BERT using the entity pair data set of on-board fault knowledge. BERT has two-parameter intensive settings, BERTlarge and BERTbase. The BERTlarge requires more memory than the BERTbase [17]. Therefore, we use BERTbase as the basic model for further processing. With a stack of 12 Transformer encoders [21], BERTbase contains 768 hidden layers and 12 self-attention heads, and the basic structure of the Transformer encoder is shown in Figure 5. The Transformer encoder is composed by two parts: a multi-headed self-attention machine and a fully connected layer in which residual connections and layer normalization operations enhance the extraction and retention of features. Due to the limited contextual information of the on-board system fault entities, it is necessary to rely on BERT's self-attention mechanism to capture the global dependencies of the sequences and learn the deep internal features of the on-board fault entity pair sequence.
The token representations of input on-board fault entity pair are first through the self-attention layer, making the model more focused on the semantic features of fault entities in various subspaces. For a sequence of fault entity pair token representations *E*={*E*_1_, *E*_2_,…, *E*_*N*_}, the attention sub-layer's output *Z*={*Z*_1_, *Z*_2_,…, *Z*_*N*_} can be calculated as [19]:
  (4) $$\begin{array}{c} a_{ij}^{\left(k\right)}=soft\max\left(\frac{1}{\sqrt{d_{Z}}}\cdot {\left(W_{Q}^{\left(k\right)}\cdot E_{i}\right)}^{T}\cdot \left(W_{K}^{\left(k\right)}\cdot E_{j}\right)\right),\\ Z_{i}^{\left(k\right)}=\sum_{j=1}^{N}a_{ij}^{\left(k\right)}\cdot \left(W_{V}^{\left(k\right)}\cdot E_{j}\right),\\ Z_{i}=W_{O}\left[Z_{i}^{\left(1\right)},Z_{i}^{\left(2\right)},\ldots ,Z_{i}^{\left(k\right)}\right], \end{array}$$
where *d*_*Z*_=*d*_*E*_/*K*, *d*_*E*_ and *K* denote the hidden states' dimension and self-attention heads' number, respectively. *W*_*Q*_^(*k*)^ ∈ *R*^*d*_*Z*_×*d*_*E*_^, *W*_*K*_^(*k*)^ ∈ *R*^*d*_*Z*_×*d*_*E*_^, *W*_*V*_^(*k*)^ ∈ *R*^*d*_*Z*_×*d*_*E*_^, and *W*_*O*_^(*k*)^ ∈ *R*^*d*_*Z*_×*d*_*E*_^ are the parameter matrices.
The transformer encoder contains optimization operations such as residual structure and layer normalization to reduce the risk of vanishing gradient and weight matrix degradation caused by the increase in depth of neural networks. Equation (5) is used to calculate the optimized output *Z*′={*Z*_1_′, *Z*_2_′,…, *Z*_*N*_′}.
  (5) $$\begin{array}{c} Z^{\prime }=layerNorm\left(E+Z\right). \end{array}$$
Input *Z*′ to the fully connected network, *H*={*H*_1_, *H*_2_,…, *H*_*N*_} can be output after calculation:
  (6) $$\begin{array}{c} H_{i}=W_{2}\cdot \text{RELU}\left(W_{1}\cdot Z_{i}^{\prime }+b_{1}\right)+b_{2}, \end{array}$$
where *W*_1_ ∈ *R*^*d*_*E*_×*d*_*E*_^, *b*_1_ ∈ *R*^*d*_*E*_^, *W*_2_ ∈ *R*^*d*_*E*_×*d*_*E*_^, *b*_2_ ∈ *R*^*d*_*E*_^ are the parameters. The output *H*′={*H*_1_′, *H*_2_′,…, *H*_*N*_′} can be calculated following residual connection and layer normalization operation.
  (7) $$\begin{array}{c} H^{\prime }=\text{layerNorm}\left(Z^{\prime }+H\right). \end{array}$$
Finally, the contextual representation of the on-board system fault entity pair sequence is generated.
(3) Output layer: Given an input on-board fault entity pair sequence *X*={*x*_1_, *x*_2_,…, *x*_*N*_}, BERT outputs the representations *H*′={*H*_1_′, *H*_2_′,…, *H*_*N*_′} of each token through the transformer encoder. In existing studies, the common approach to solving sentence matching tasks utilizing BERT models is to add a token [CLS] at the beginning of the input sequence and use the state vector of [CLS] at model's last layer to represent the features of the whole input sequence [16, 22]. The fully connected network is usually joined after the last layer of tokens [CLS], and then Softmax is used to combine all the extracted features for sentence pair matching. The entity pair matching of train control on-board fault knowledge is a pairwise binary classification task. The key information of the on-board fault entity needs to be captured by BERT, so the feature extraction ability of fault knowledge is very important. To investigate the effectiveness of the features selected by BERT from different layers in the on-board fault knowledge fusion task, three output structures present for fault EM are proposed, inspired by the work of Refs. [18–20].  Figure 6 illustrates the three models' output structures. By incorporating the Softmax layer on each output structure's bottom, we can calculate conditional probability distributions on the predefined fault entity pair categorical labels.
  (i) The token representations that correspond to [CLS] of BERT's last layer are used to represent the whole sequence and connect it to the Softmax classifier, which is recorded as BERT_*LAST*_.
  (ii) Connect the token representations corresponding to [CLS] of each layer in the last four layers of BERT as the representation of the whole sequence, and then sent to the Softmax classifier, which is recorded as BERT_*CON*_.
  (iii) Take the token representations corresponding to [CLS] in the second last layer of BERT used to represent the whole sequence, and connect it to the Softmax classifier to predict the conditional probability distributions of labels, which is recorded as BERT_*SEC*_.

### 4.3. Model Pretraining and Fine-Tuning Strategy

The training of the on-board system fault knowledge fusion model on BERT is divided into two stages: first, it is pretrained using large unlabeled corpora, and then it is fine-tuned by supervised learning to achieve entity pairs matching of the train control on-board KB. The unsupervised pretraining process is implemented by BERT using the MLM and NSP methods. The former uses the MLM method to predict arbitrarily masked words, while the latter is used to judge if the input sentence is consecutive. Meanwhile, BERT provides a pretrained model for Chinese.

Once it has been pretrained, BERT can be fine-tuned for downstream tasks using supervised learning once it has been pretrained to make it more suitable for the specific domain task of matching for train control on-board system fault knowledge units. Therefore, this paper will investigate the fine-tuning methods of the BERT-based model for EM of train control on-board system fault knowledge, including output structure selection, semi-supervised incremental training strategy based on pseudo-label, and the adversarial training based on embedding.

The first is the output structure selection of the BERT-based model for EM. Since different hierarchical structures in BERT extract different syntactic or semantic features for on-board fault knowledge entity pairs, in order to make BERT adapt to our task, three different output structures are designed to obtain the output features of BERT in the fine-tuning, so as to select the most effective hidden layer features for heterogeneous knowledge fusion. For the EM task oriented to knowledge fusion, it is necessary to judge whether the input two entities point to the same object. In this paper, the matched entity-pair is labeled as 1, and the unmatched entity-pair is labeled as 0. All parameters in BERT are jointly updated by maximizing the conditional probability of correct labels.

**Figure 7:**
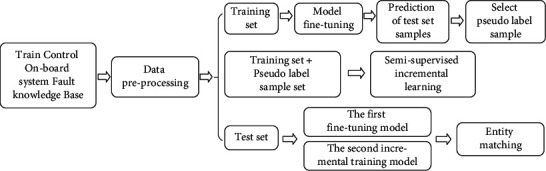
SSI learning strategy based on pseudo label.

In the EM task for fault knowledge fusion of high-speed railway on-board system, the training, validation, and test sets are constructed. Since there is a huge volume of on-board fault knowledge, if all entities in the fault KB are matched and labeled in pairs, the cost of construction is relatively high. The test set includes a great number of unlabeled samples, the hidden information is not fully utilized by the model. In most supervised learning methods, a considerable amount of labeled data is required to train a model. However, semi-supervised learning methods can utilize unlabeled data to train the model. In semi-supervised learning, graph-based and pseudo-label-based methods are typically included according to the theoretical basis. The graph-based methods involve building a nearest-neighbor graph, assuming that the connected nodes are similar and have the same labels, and learning about the distributions of data structures and categories from the unlabeled samples, which is a feature ranking algorithm [23]. Pseudo-label-based methods utilize labeled data to train single or integrated classifiers, which expand the supervised data set with the pseudo label generated by the classifier, and it is an iterative “prediction-selection” process [24]. To decrease the construction burden of labeled data sets, maximize the advantages of unlabeled and limited-labeled samples, and rapidly expand the size of the training set, we propose a pseudo-label-based semi-supervised incremental (SSI) learning strategy, which enables the model to collaboratively utilize pseudo-labeled samples to further optimize the heterogeneous knowledge fusion effect. Although unlabeled test data does not have the label information, they are the same as labeled data, which are obtained from the same data source and meet the assumption of independent and identically distributed [25]. Therefore, the information they contain is very beneficial to the optimization model. The SSI learning strategy is shown in Figure 7. In the first stage, the EM model is fine-tuned with the labeled training set until the training result reaches the expected accuracy, and the supervised training is stopped. Then pseudo labels are generated. The unlabeled fault entity pairs set is predicted using the EM model created in the first stage, and the probability value output by the Softmax layer is used as the evaluation index to judge the confidence of the fault EM model to the prediction labels. The prediction label of fault entity pairs with high confidence is taken as the pseudo label, and the pseudo label data are added to the training set for incremental learning. In this step, the labeled data and pseudo labeled data are combined to train the model for the second time. In the process of supervised training and incremental training, the validation set remains unchanged. When finally predicting the test set, the prediction label can be jointly output according to the output results of the first fine-tuning model and the output results of the second training model to complete the final entity pair matching of on-board heterogeneous knowledge units.

Studies have shown that most neural networks are very sensitive to changes in input, and small input disturbances may cause large output differences [26]. In semi-supervised learning, which is prone to the issue of insufficient generalization ability caused by random noise, some wrong entity pairs will inevitably be introduced when adding pseudo-label samples to the on-board system fault entity pair training set. The input of noise data will cause the model to output incorrect answers with high credibility, which is not conducive for the learning quality of the EM model. To overcome the negative effect of random noise similar to adversarial samples on the model performance and further enhance the generalization ability and robustness of domain EM models for on-board system fault knowledge, this work introduces adversarial training based on embedding as a regularization strategy. The basic idea of adversarial training is to construct adversarial samples to attack the trained network in order to adjust the network parameters to improve robustness so that the network can resist these attacks [27]. We add a small disturbance to the input embedding to generate antagonistic data, and the antagonistic data and the original sample are used as the inputs of the fault knowledge EM model. Adversarial training refers to calculating the disturbance in maximizing the loss and minimizing the loss of the model after increasing the disturbance. When perturbations are introduced into the input embedding of the EM model, it is necessary to introduce disturbance parameters into the loss functions [28], that is:

(8) $$\begin{array}{c} L=-\log\;p\left(y|x+r_{adv};\theta \right), \end{array}$$

(9) $$\begin{array}{c} r_{adv}=\underset{r,\left\|r\right\|\leq \varepsilon }{\arg\min\;\log}p\left(y|x+r;\overset{⌢}{\theta }\right), \end{array}$$

where *x* is the input and *θ* are the parameters of the model, *r* is a perturbation on the input embedding, $\overset{⌢}{\theta }$ is a constant set to the current parameters. In each step of training, the most influential perturbations *r*_*adv*_ is generated for the current model in (9), and then the model is trained by minimizing (8) with respect to *θ* to defend against error perturbations and finding the model parameters with the highest robustness. However, we cannot accurately calculate the input perturbations *r*_*adv*_ in (9). Therefore, Goodfellow et al. [27] proposed a linear approximation method shown in (10) and (11) with norm constraints to obtain the adversarial perturbation.

(10) $$\begin{array}{c} r_{adv}=-\frac{\varepsilon \cdot g}{{\left\|g\right\|}_{2}}, \end{array}$$

(11) $$\begin{array}{c} g=\nabla_{x}\log\;p\left(y|x;\hat{\theta }\right). \end{array}$$

This approximate method can obtain the input perturbations more easily, and then optimize the model parameters through (8) to strengthen the EM model's robustness and optimize the effect of heterogeneous knowledge fusion.

## 5. Experiments

### 5.1. Data Sets and Experimental Settings

**Figure 8:**
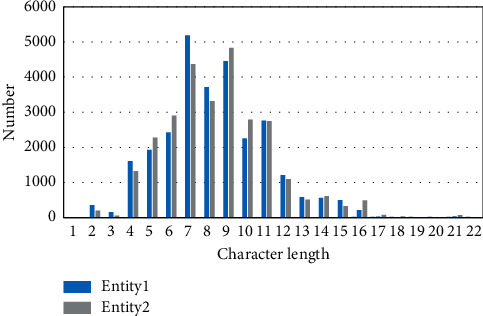
Character length distribution of entities.

Based on the fault knowledge base of the on-board system, the goal of this paper is to realize the fusion of heterogeneous knowledge units, the elimination of semantic conflict, and the unified integration of multi-source information through EM. To examine our model's performance in the heterogeneous knowledge fusion task of the train control on-board system, this paper constructs the fault KB based on the fault maintenance data accumulated from 2017 to 2021 of the CTCS-3 train control on-board system operating on the Xuzhou-Lanzhou High-speed Railway line. On the basis of fault KB, the entity pairs data set of on-board fault knowledge is constructed according to the method proposed by data preprocessing. We chose six types of entities for the knowledge fusion task in this experiment, including fault type, fault cause, fault analysis, equipment phenomenon, treatment measures, and maintenance measures. In the sample set, there are 26000 samples of which the ratio of positive samples (labeled as 1) to negative samples (labeled as 0) is approximately 45% to 55%. Count the character length of entity 1 and entity 2 contained in the entity pair.  Figure 8 depicts the entities' character length distribution. The length is mainly between 2 and 22, which is a typical sparse short text. A ratio of 6 : 2 : 2 is used to separate the data set into three parts for training, validation, and testing.

The computer configuration for this experiment is an Intel(R) Core(TM) i7-10750H processor with 2.6 GHz and an NVIDIA GeForce RTX 2060 GPU. All models are implemented in the Python programming language. The BERT-based EM model is encoded using BERTbase, and the dropout rate has been set to 0.2 on the fully connected layer to prevent overfitting of the model. By adjusting the parameters of the model, we adjust the batch size and epochs to 24 and 10, respectively, and select an Adam optimizer with a learning rate of 1*e* − 5 to train the model and introduce early stops during the training process.

### 5.2. Contrast Model

To comprehensively evaluate the effectiveness of the EM model in the heterogeneous knowledge fusion task of the on-board system, three metrics including macro-precision (Marco-P), macro-recall (Marco-R), and macro-F (Marco-F) are used for experimental evaluation. And compare our model with other solutions developed for related matching tasks:

  LSTM-Siamese [29]: The model incorporates a character-level bi-directional LSTMs with a Siamese network to match semantics between text pairs. We use the Manhattan distance to calculate the semantic similarity between strings to increase the matching accuracy.
  ABCNN-3 [30]: In this work, the attention structure is introduced into a convolutional neural network (ABCNN) to construct a sentence pair matching model. ABCNN-3 constructs the attention matrix on both the convolution and pooling parts to establish the connection between sentence pairs.
  BIMPM [31]: This paper presents a BiMPM model in which two sentences are encoded by a BiLSTM encoder, and the encoded sentences are matched in two directions. Then, another BiLSTM can produce matched results.
  ESIM [32]: A chain LSTM-based inference model has been designed, and the recursive architecture in both local inference modeling and inference composition is considered.
  BERT [17]: This is a basic BERT model. The token representations that correspond to [CLS] of BERT's last layer are used to represent the whole sequence.
  ALBERT [33]: It is a lightweight variant of the BERT structure. To reduce memory consumption and improve training speed, two-parameter reduction technologies are proposed, namely factorized embedding parameterization and self-supervised loss for sentence-order prediction.
  ERNIE [34]: Based on the idea of token-entity alignments masking, an enhanced model is trained using a large-scale text corpus and knowledge graph.

### 5.3. Model Comparison Results

Table 2 summarizes each model's results for the heterogeneous knowledge matching task of the high-speed railway on-board system. Part I are semantic similarity methods, which focus on binary similar entity pair matching by embedding-based models. Part II and III focus on constructing knowledge matching models through various network designs based on BERT, with Part II involving earlier BERT-based variants, and Part III involving different output structures.

Each model's test results are presented in Table 2. The AD-BERT_*LAST*_-SSI model achieves the optimal effect in knowledge fusion of train control on-board system fault KB, and outperforms the strongest baseline model ERNIE by 1.72% for the Marco-R and 1.65% for the Macro-F. According to the experimental results, it can be found that the enhancement of the knowledge fusion effect is primarily for several reasons:

Encoder: In Part I of the experiment, ESIM obtained the best performance in this part, and the Macro-F reached 0.9086. This model makes use of the good sequence modeling ability of LSTM, obtains the local semantic relationship between entity pairs in on-board knowledge units through an attention mechanism, and integrates the local information to construct global reasoning. The methods in Part II and III are better than all embedding-based models in heterogeneous knowledge fusion, and ALBERT is 4.43% higher than the Macro-F of ESIM. The models in Part II and Part III make full use of the advantages of BERT, which constructs a deep network by stacking bi-directional transformer encoders. In comparison with LSTM, this construction method is able to extract local and global dependencies between the on-board entity vocabulary and the context more deeply through self-attention techniques. The multi-level structure of BERT can obtain higher quality semantic and syntactic features, which makes a good foundation for semantic matching of on-board heterogeneous knowledge.

Language Model Pretraining: In the task of heterogeneous knowledge fusion in the on-board system, the BERT-based EM models fully utilize the pretrained model and thus outperform the embedding-based models in fusion. In the knowledge fusion method based on semantic similarity, words need to be converted into low-dimensional vector space using an embedding matrix, and then features are extracted from the on-board knowledge units by deep networks. The parameters of the model are randomly initialized and need to be learned from scratch, so such models do not benefit from pretraining. The model based on pretraining combined with fine-tuning is more flexible. The BERT can be fine-tuned directly to complete the fusion of heterogeneous knowledge.

Fine-tuning Strategies: The experimental results of Part III are better than those of Part II. After the adversarial training and SSI learning strategies are integrated into the BERT model with three output structures, the Macro-F of knowledge fusion is higher than that of ALBERT and ERNIE. By contrast, the combination of SSI learning in BERT can effectively use the semantic feature information of on-board entities contained in unlabeled samples to enhance the model's generalization ability, as well as by using adversarial training to enhance the model's robustness, so as to improve the effect of EM. Through the experimental verification, it can also be seen that the feature from BERT's last layer gives the best performance, which is 0.18% higher than the Macro-F of AD-BERT_*SEC*_-SSI and is more useful for heterogeneous knowledge fusion.

### 5.4. Ablation Study

We conducted ablation experiments from the perspective of adversarial training and SSI learning strategies to remove some components from the AD-BERT_*LAST*_-SSI, AD-BERT_*CON*_-SSI, and AD-BERT_*SEC*_-SSI proposed in this paper, and judged the contribution of each component in the heterogeneous knowledge fusion task of the on-board system according to the performance (Tables 3to 5).

The core part of the proposed method is a BERT-based heterogeneous knowledge fusion model, which introduces adversarial training and SSI learning strategies based on the three output structures of the model. Firstly, it can be seen from the experimental results that the BERT-based knowledge fusion model achieves excellent results after removing adversarial training and SSI learning in the presence of abundant data in the training set. Even the basic BERT_*CON*_ has a Marco-F of 0.9784. Through the ablation experiment, we further observe the general improvement effect of each optimization strategy on the BERT-based model, so as to judge its contribution.

When removing the adversarial training from the three EM models of AD-BERT_*LAST*_-SSI, AD-BERT_*CON*_-SSI, and AD-BERT_*SEC*_-SSI, the Marco-F of on-board system heterogeneous knowledge fusion of the three models showed an overall decrease of some magnitude, and the maximum decreased by 0.54%, which appears on AD-*BERT*_*CON*_-SSI. The general degradation of model performance is due to the existence of unlabeled samples in incremental learning, and the data enhancement method with random noise is not conducive to semi-supervised learning. After using adversarial training to generate adversarial noise that is more relevant to the on-board knowledge, the ability of the model to identify input disturbance is improved, which can facilitate the model to achieve the purpose of identifying unknown samples. Therefore, the experimental results show that after removing the adversarial training, the fusion effect of the models on heterogeneous knowledge all showed a decrease. The adversarial training based on embedding strengthens the EM model's resolution ability by input disturbances, as well as its generalization ability and robustness in the on-board system's heterogeneous knowledge fusion.

When removing the SSI, the EM models cannot fully utilize the unlabeled samples, resulting in the decline of knowledge fusion performance of the three models to a certain extent, and the precision and recall are generally decreased. Among them, the performance decreases the most after AD-BERT_*SEC*_-SSI removes SSI, with a decrease of 0.23% in Marco-F. In this work, a combination of the BERT and semi-supervised learning is used. The former has a deeper network structure, and can obtain more detailed feature information of entity pairs in on-board system knowledge units through pretraining and fine-tuning. This model has achieved high accuracy in the supervised training stage, which also provides a reliable basis for the generation of pseudo labels. The latter increases the incremental learning based on unlabeled test samples, expands the training set's scale, and makes full use of pseudo-label samples to further enhance the ability of the EM model in knowledge fusion. Certainly, the number of labeled samples also directly affects the fusion effect of the supervised model, and we continue to verify it in the next section of the experiment.

When both adversarial training and SSI learning are removed, the maximum decrease of Marco-F is 0.73%, which appears on AD-BERT_*CON*_-SSI. The heterogeneous knowledge fusion of the basic BERT model is already powerful after fine-tuning the model through supervised learning using the knowledge base. Among the proposed optimization strategies, adversarial training and SSI learning are synergistic with each other. When the two optimization strategies are incorporated into the models separately, the Marco-P, Marco-R, and Marco-F of the three models on the on-board system heterogeneous knowledge fusion task are further improved to various extents, and the improvement is stable, so the addition of these components is necessary.

### 5.5. Analysis of SSI Learning Strategy

**Figure 9:**
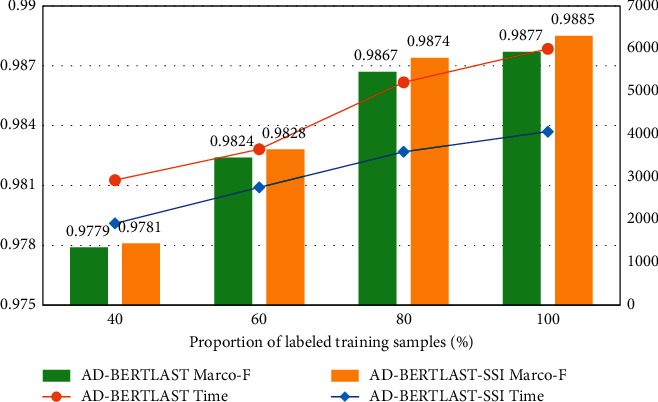
Experimental results under different labeled sample numbers.

Using a limited number of supervised on-board fault entity pairs data set to fine-tune the BERT in downstream tasks is crucial. To investigate the influence of the proposed model and semi-supervised incremental learning strategy on on-board heterogeneous knowledge fusion under different numbers of labeled samples, we carried out experimental verification under varying numbers of labeled data. Some data from the training data are selected as labeled training data in proportions of 40%, 60%, 80%, and 100% to produce four separate training sample sets, while the validation set and test set remain unchanged. Because AD-BERT_*LAST*_-SSI has the best performance among the three EA models proposed, take this model as an example, take AD-BERT_*LAST*_ as the comparison model to verify the effect of on-board system heterogeneous knowledge fusion.  Figure 9 displays the Marco-F values and each Epoch's training time(s) for the two models on different scales of the training set, where the AD-BERT_*LAST*_ shows the average fine-tuning time of each Epoch. Since AD-BERT_*LAST*_-SSI is incremental learning on AD-BERT_*LAST*_, the model shows the training time of the first round of incremental learning.

The BERT base model includes a huge number of parameters, about 110M, so the fine-tuning process of the AD-model is also very time-consuming. As illustrated in Figure 9, the model fine-tuning time and incremental learning time grow as the data set gradually expands. The SSI strategy requires several rounds of iterative learning, and as the number of iterative rounds increases, the total training time also increases accordingly round by round.

From the Marco-F value, the fusion effect of the on-board fault knowledge of the two models gradually improves with the growth of the labeled sample size. As the training sample size grows, more prior information is contained in the training set, and the Marco-F of the two EM models will also increase.

AD-BERT_*LAST*_ is a supervised training model. When using only 40% of the labeled data for training, the Marco-F for the on-board system heterogeneous knowledge fusion task is 0.9779, which is 0.98% lower than when using 100% of the data for training. It shows that the supervised learning method is very dependent on the label information in the training samples, and when the label information is insufficient, the effect of knowledge fusion will be significantly reduced.

AD-BERT_*LAST*_-SSI is a semi-supervised incremental learning method. Under the proportion of four kinds of label samples, the result of on-board fault knowledge fusion of AD-BERT_*LAST*_-SSI is better than that of AD-BERT_*LAST*_. Even when the number of labeled samples is only 40%, the AD-BERT_*LAST*_-SSI model can obtain a better matching effect for entity pairs than the supervised training model, and the Marco-F value is increased by 0.02%.

It shows that when the supervised training model can better fuse the heterogeneous knowledge of the on-board system, combined with the semi-supervised incremental learning method, it can accurately expand the labeled samples, and reduce the dependence of the DL model on the label data to a certain extent. When labeled data are limited, the SSI method also has good advantages in the heterogeneous knowledge fusion task of the on-board system.

In practical applications, the scale of labeled data can be controlled to make the DL model have sufficient sample size support. On the other hand, supervised models can be used as pseudo-labeled sample generators to promote data utilization and knowledge fusion by incremental learning of unlabeled samples. This means that SSI learning is capable of reducing the construction cost of the entity pairs data set of on-board fault knowledge, improving the fusion effect of heterogeneous knowledge units, and has strong application value.

## 6. Conclusion

Processing multi-source heterogeneous O&M data of high-speed railway on-board system and building a high-quality fault knowledge base are important foundations to support knowledge-driven dynamic O&M decision-making of the on-board system. To solve the problem of multi-source heterogeneity of fault knowledge, this work proposes a semi-supervised incremental learning EM model based on BERT to complete the knowledge fusion task. After detailed experimental verification, the results showed that:

1. The BERT-based EM model can effectively capture the deep semantic information hidden by knowledge units in the absence of contextual information. The feature from the BERT's last layer gives the best performance, which is more useful for the heterogeneous knowledge fusion task of the on-board system.
2. A pseudo-label-based SSI learning strategy is introduced based on the EM model to enable the model to collaboratively utilize pseudo-labeled samples to strengthen the model's generalization capability. This method reduces the dependence of the model on labeled data and has advantages when labeled data are limited.
3. Combining the model with adversarial training, adversarial training over noise data and clean data is adopted to update the parameters of the model, guiding the model to learn noise-independent hidden representations, boosting the model's robustness and enhancing the effect of heterogeneous knowledge fusion of the on-board system.

## Figures and Tables

**Table 1:** Examples of entities in the on-board system fault KB.

Data type	Entity 1	Entity 2
Fault type	A-Kernel mode transition invalid	A mode transition invalid
Equipment phenomena	CTCS-3 exception downgraded to CTCS-2	C3 ⟶ C2
Fault cause	Wireless communication connection timeout	Infinite communication connection timeout
Treatment measure	Switching and restarting the system	Change system and reboot
Maintenance measure	Replace emergency brake relay	Check emergency brake relay

**Table 2:** Experimental results under different EM models.

	Entity representation	Models	Marco-P	Marco-R	Marco-F
I	Embedding-based models	ABCNN-3	0.8782	0.8762	0.8765
		LSTM-Siamese	0.8553	0.8219	0.8374
		BIMPM	0.9078	0.8998	0.8985
		ESIM	0.9127	0.9093	0.9086

II	BERT-based models	ALBERT	0.9553	0.9511	0.9529
		ERNIE	0.9729	0.9711	0.9720
III		AD-BERT_*LAST*_-SSI	0.9887	0.9883	0.9885
		AD-BERT_*CON*_-SSI	0.9859	0.9856	0.9857
		AD-BERT_*SEC*_-SSI	0.9871	0.9863	0.9867

**Table 3:** Ablation study on AD-BERT_*LAST*_-SSI Model.

Model	Marco-P	Marco-R	Marco-F
AD-BERT_*LAST*_-SSI	0.9887	0.9883	0.9885
*Remove components*			
adversarial training	0.9874	0.9868	0.9871
SSI learning	0.9879	0.9875	0.9877
adversarial training & SSI learning	0.9860	0.9857	0.9859

**Table 4:** Ablation study on AD-BERT_*CON*_-SSI Model.

Model	Marco-P	Marco-R	Marco-F
AD-BERT_*CON*_-SSI	0.9859	0.9856	0.9857
*Remove components*			
adversarial training	0.9812	0.9796	0.9803
SSI learning	0.9851	0.9850	0.9851
adversarial training & SSI learning	0.9794	0.9776	0.9784

**Table 5:** Ablation study on AD-BERT_*SEC*_-SSI Model.

Model	Marco-P	Marco-R	Marco-F
AD-BERT_*SEC*_-SSI	0.9863	0.9871	0.9867
*Remove components*			
adversarial training	0.9871	0.9861	0.9866
SSI learning	0.9845	0.9842	0.9844
adversarial training & SSI learning	0.9839	0.9838	0.9838

## Data Availability

The data used to support the findings of this study are available from the corresponding author upon request.
